# Digital Physical Activity and Sedentary Behavior Interventions for Community-Living Adults: Umbrella Review

**DOI:** 10.2196/66294

**Published:** 2025-03-18

**Authors:** Eilidh Russell, Alison Kirk, Mark D Dunlop, William Hodgson, Mhairi Patience, Kieren Egan

**Affiliations:** 1 Department of Computer and Information Sciences University of Strathclyde Glasgow United Kingdom; 2 Department of Physical Activity and Health University of Strathclyde Glasgow United Kingdom

**Keywords:** physical activity, sedentary behavior, digital health interventions, behavior change, theoretical frameworks, umbrella review, mobile phone, community-living adults

## Abstract

**Background:**

Digital interventions hold significant potential for improving physical activity (PA) and reducing sedentary behavior (SB) in adults. Despite increasing interest, there remain surprising gaps in the current knowledge of how best to deliver these interventions, including incorporating appropriate theoretical frameworks and behavior change techniques. Following numerous systematic reviews, there is now significant potential for umbrella reviews to provide an overview of the current evidence.

**Objective:**

This umbrella review aimed to explore digital PA and SB interventions for community-living adults across effectiveness, key components, and methodological quality.

**Methods:**

This review followed the Joanna Briggs Institute framework for umbrella reviews. Key search terms were developed iteratively, incorporating physical and sedentary activity alongside digital interventions. We searched 7 online databases (Web of Science Core Collection, CINAHL, APA PsycINFO, Inspec, the Cochrane Library, MEDLINE [Ovid], and PROSPERO) alongside gray literature databases. Information was extracted and tabulated from each included article on intervention effectiveness, key components, and content acknowledging both the digital and human elements. The study quality was appraised using A Measurement Tool to Assess systematic Reviews 2 (AMSTAR 2). The corrected covered area method was used to assess the overlap of primary studies included in the systematic reviews. All relevant research findings were extracted and reported.

**Results:**

Search terms identified 330 articles, of which 5 (1.5%) met the inclusion criteria. The most common PA outcomes identified were daily steps, moderate-to-vigorous PA, total PA, and PA change. Reviews with meta-analysis reported that digital interventions improved multiple PA outcomes (daily steps, moderate-to-vigorous PA time, and total PA time). However, findings from the remaining systematic reviews were mixed. Similarly, the findings for SB were contrasting. Regarding intervention components, monitor- and sensor-only intervention delivery methods were most frequently implemented. Eleven theoretical frameworks were identified, with social cognitive theory being the most prominent theory. In total, 28 different behavior change techniques were reported, with goal setting, self-monitoring, feedback, and social support being the most frequently used. All 5 systematic reviews were of low or critically low quality, each incorporating unique primary studies (corrected covered area=0%).

**Conclusions:**

This umbrella review highlights the potential of digital interventions to increase PA and reduce SB among community-living adults. However, the disparate nature of current academic knowledge means potentially efficacious research may not realistically translate to real work impact. Our review identified a lack of consensus around outcomes and components at both individual (eg, difficult to collate and compare findings) and multiple study (poor reported quality of systematic reviews) levels. Collective, concerted action is required to standardize outcomes and improve systematic review reporting to optimize future learning around digital interventions to increase PA and reduce SB in community-living adults, including traditionally overlooked populations, like informal carers.

**Trial Registration:**

PROSPERO CRD42023450773; https://www.crd.york.ac.uk/PROSPERO/view/CRD42023450773

## Introduction

### Background

Physical activity (PA) is defined as “any body movement generated by the contraction of the skeletal muscles that raises energy expenditure above resting metabolic rate and is characterized by its modality, frequency, intensity, duration, and context of practice” [1]. Engaging in regular PA can reduce the risk of cardiovascular disease, type 2 diabetes, cancer, weight gain and obesity, and Alzheimer disease and significantly improve mental health and quality of life [2-4]. Alongside these individual health benefits, PA also provides benefits for societies, environments, and economies [5]. Sedentary behavior (SB) is described as “any waking behaviors characterized by an energy expenditure ≤1.5 metabolic equivalents while in a sitting, reclining, or lying position” [1]. Prolonged SB has been found to increase all-cause mortality and is also associated with poor disease outcomes, such as increased risk of developing cardiovascular disease, diabetes mellitus, hypertension, and certain types of cancer [6-8].

Despite the well-documented evidence for improving PA levels and reducing SB, many adults do not engage in sufficient levels of activity and have high levels of SB. Data from the Lancet assessing global trends in insufficient PA among adults from 2000 to 2022 using population-based surveys found that nearly one-third (31.3%) of the adults worldwide were not meeting the World Health Organization recommendations for moderate-to-vigorous aerobic activity in 2022 [9]. Findings from this work also suggest that if trends from 2010 to 2022 continue, the global prevalence of insufficient PA will reach 34.7% by 2030. Therefore, there is a need for effective interventions to increase PA levels and reduce SB.

Digital health interventions (DHIs) can be used to promote PA and reduce SB. World Health Organization describes digital health as the use of digital, mobile, and wireless technologies to aid the achievement of health-related objectives and includes both mobile health and eHealth [10]. DHIs can include smartphone apps, wearable activity trackers, online platforms, and web-based interventions. A favorable feature of DHIs is their ability to be scaled for large populations while also being able to be tailored for specific populations [10]. The use of DHIs has also been recommended by the global action plan on PA 2018 to 2030 to promote and support participation in PA due to their success and benefits of use [11].

As PA is described to be a complex behavior [12,13], it requires more time and commitment to change habits than other health behaviors. It has been recommended that PA and SB interventions be informed by theoretical frameworks and include appropriate behavior change techniques (BCTs) as they can be important for increasing effectiveness and adherence toward the behaviors [14,15]. Despite such previous work, there is little consensus as to what the best theoretical frameworks and BCTs for changing PA and SB are. For example, research suggests that combined PA and dietary interventions based on specific theories, such as cognitive theory and theory of planned behavior, are no more effective in achieving changes to PA outcomes than interventions with no stated theoretical grounding [16]. However, meta-regression of this work and an additional study [17] suggest that the use of a cluster of “self-regulatory” intervention techniques had a positive relationship with increasing PA, alongside additional outcomes, such as weight loss and dietary changes. Similarly, the available evidence to support the use of BCTs to improve PA is contrasting, with positive changes to PA behavior reported, but not all findings were statistically significant. Therefore, digital interventions with a theoretical grounding and BCTs could encourage improvements in PA and SB; however, more investigation into these aspects of intervention is required.

Moreover, many groups in society face significant additional barriers to PA either in an acute or chronic perspective, including individuals who face mental and physical health conditions, those facing higher levels of economic deprivation, and individuals in later years of life. An emerging and sizable group who are facing systemic barriers to PA and reducing SB includes informal carers. Informal carers could particularly benefit from digital PA and SB interventions as Horne et al [18] found that carers often prioritize the needs of those they care for over their own health, which could result in lower levels of PA and higher levels of SB. Caring duties can also act as a barrier for where, when, and how long they can participate in PA programs. Allowing this group a digital intervention that considers their own unique circumstances, preferences, and barriers could significantly improve adherence to higher PA levels and reduce SB. There is little published evidence to understand the use of these digital interventions for improving these behaviors within this population. In contrast, there are numerous primary studies and systematic reviews exploring the use of digital interventions to improve PA and SB levels in adults. Although having numerous systematic reviews on a certain topic can be beneficial, it has been reported that too many findings on a specific topic or research area can lead to replication issues by amplifying biases, inconsistencies, and misinterpretations from the available literature [19]. To eliminate the issues caused by multiple systematic reviews, umbrella reviews have been recommended.

Umbrella reviews are used to summarize the evidence from multiple research syntheses. This type of review provides an overview of the available evidence on a chosen topic and can be used to compare or contrast findings from published systematic reviews [20]. An umbrella review is particularly beneficial for decision makers as it provides “user-friendly” summaries of the available knowledge and literature without the need to read and understand the findings of multiple systematic reviews themselves [21]. Umbrella reviews evaluating digital interventions currently do exist [22-24]. However, only a few specifically focus on groups within society who face additional barriers to participating in PA.

### This Study

The overall scope of this research is to support PA and reduce SB in informal carers via digital devices. However, the lack of published primary studies and systematic reviews exploring digital PA and SB interventions for informal carers means that this paper will focus on digital PA and SB interventions for the community-living adult population, as there are numerous published systematic reviews in this topic area. We will address, in the Discussion section, the potential implications for the caring population.

The research questions for this umbrella review were as follows:

1. How effective are digital interventions in changing PA and SBs in community-living adults?
2. What components are included in digital PA and SB interventions, including delivery methods (technologies and human elements used) and content (theoretical frameworks and behavior change strategies used)?
3. What is the methodological quality of the included systematic reviews?

## Methods

The methodology for this umbrella review was guided by the Joanna Briggs Institute Manual for Evidence Synthesis [25]. A protocol for this review was submitted to PROSPERO (CRD42023450773).

### Inclusion Criteria

The inclusion criteria were generated using the population, intervention, context, outcomes, and study (PICOS) method. The inclusion and exclusion criteria for this umbrella review are shown in Textboxes 1 and 2.

**Inclusion criteria.**

**Population**

- Community-living adults aged ≥18 years.

**Interventions**

- Digital interventions that were designed to increase physical activity (PA) levels and reduce sedentary behavior (SB) in adults who are physically capable of living at home.
- Interventions could be multimodal in nature (eg, they could include a digital and in-person element) if the digital element was the main component of the intervention.

**Context and outcomes**

- The main outcomes of this review were the effectiveness of interventions in changing PA and SB.
- The components and content included in digital PA and SB interventions, including the technological and human components, behavior change strategies, and theoretical frameworks, were also the main outcomes of this review.
- The overlap of included primary studies and the quality of the systematic reviews were secondary outcomes of this umbrella review.

**Study type**

- Systematic review.

**Publication date**

- Published in or after 2013.

**Language**

- English.

**Exclusion criteria.**

**Population**

- Children or young adults aged ≤18 years.

Adults who were not capable of living independently or not community living.

**Interventions**

- Digital interventions that were not designed to increase PA levels and reduce SB.

Interventions where the digital element was not the main component of the intervention.

Digital interventions targeting multiple behaviors (eg, diet and PA).

**Context and outcomes**

- Systematic reviews of digital PA and SB interventions that do not report on the included outcomes (eg, systematic reviews assessing the effectiveness of PA and SB interventions on reducing blood glucose or mental health outcomes).

**Study type**

- Primary studies or any other type of review.

**Publication date**

- Published before 2013.

**Language**

- Other than English.

### Search Strategy

Searches were conducted in July 2023 in the following 7 chosen databases: Web of Science Core Collection, CINAHL, APA PsycINFO, Inspec, Cochrane Library, MEDLINE (Ovid), and PROSPERO. These databases were used as they contain papers from health, social care, psychology, and computing and therefore encompass the topic area of this umbrella review. The gray literature databases, such as arXiv, Core, OpenGrey, and OpenDissertations were, also searched alongside the reference list of included systematic reviews. The rationale for focusing on systematic reviews from 2013 onward allowed the research team to maximize the availability of knowledge around digital interventions for improving PA and SB. The publication period up to 2013 marked the period when digital tools became more commercial, sophisticated, and widespread. Before 2013, digital interventions often relied on less accessible or less integrated technology. However, from 2013 the advancement of sensors and integration within digital interventions became more mainstream and transformed the landscape of these interventions. The inclusion criteria allowed this review to capture technologies most relevant to the present day and the future. In addition, by including systematic reviews from the past 10 or more years, primary studies 10 years before 2013 were able to be captured.

With guidance from subject librarians, a comprehensive keyword search strategy was created. These keywords in relation to adults, digital, PA, SB, theoretical models, BCTs, and systematic reviews were all initially searched individually to help refine the search criteria and formulate a more concise and comprehensive search strategy. The final search combined all keywords to identify systematic reviews on digital PA and SB interventions for community-living adults that incorporated elements of BCTs and theoretical frameworks (Multimedia Appendix 1).

### Study Selection

Results from the database and gray literature searches (excluding Core) were imported into the EndNote Online reference manager (Clarivate). Duplicates were then removed in EndNote. Core search results were unable to be exported into this system; therefore, screening of these articles remained in the CORE database.

After this removal process, the remaining articles were exported from EndNote to the Rayyan (Rayyan Systems, Inc) website [26] for further screening. Title and abstract screening was conducted in Rayyan independently by the main reviewer (ER) and a second reviewer (WH). Title and abstract screening was blinded to reviewers until the process had been completed to lessen the potential for bias to occur. Full-text screening was also conducted in Rayyan with a supplementary Microsoft Excel document used by the main reviewer for tracking progress, dividing the workload between additional reviewers, and for additional comments and reasoning for inclusion or exclusion. Full-text screening was conducted independently by the main reviewer alongside the second reviewer (WH) and an additional reviewer (MP). The full-text screening process was also blinded. Conflicts were resolved through discussion between the reviewers.

### Corrected Covered Area

The overlap of the primary studies included in the chosen systematic reviews was also assessed. As systematic reviews with similar research questions may include some of the same studies within their reviews, it is important to measure the overlap of these studies as it can disguise a lack of current research or give a false impression of new evidence.

The corrected covered area (CCA) was calculated by creating a citation matrix where primary publications were listed in rows and the included systematic reviews of the umbrella review were represented in columns. The number of times a given study was cited in the systematic reviews was then calculated using the following formula [27]:

*CCA = N* – *r / rc* – *r*

where *N* is the total number of times primary publications appeared in reviews (inclusive of double counting), *r* is the number of unique primary publications, and *c* is the number of systematic reviews included in the umbrella review. The degree of overlap was then classified, where 0% to 5% was considered “slight overlap,” 6% to 10% was considered “moderate overlap,” 11% to 15% was considered “high overlap,” and >15% was considered “very high overlap” [27].

### Data Extraction

In order to answer research questions 1 and 2 of this umbrella review, a Microsoft Excel spreadsheet for data extraction was created using the Joanna Briggs Institute Manual for Evidence Synthesis data extraction guidelines. Extracted information included article and publication information (author, date, and digital object identifier), study information (number of included studies, date range, study design, location, and inclusion of meta-analysis), population demographics (sample size, age, and sex), intervention details, measures of effectiveness, and methodological quality rating. Data extraction was also quality checked for agreement between members of the review team.

Information on changes in PA and SB was extracted to answer the first research question. The most common PA outcomes (daily step count, moderate-to-vigorous PA [MVPA] min, total daily or weekly PA time, and PA change) and SB outcomes (SB min or change in SB) were used to report the effectiveness of these behaviors. To report on the components included within digital PA and SB interventions, the extracted information consisted of the following: whether the intervention focused on PA or SB only or if it targeted both behaviors, whether the intervention was shaped by a theoretical framework and what the frameworks were, and if the intervention included BCTs and what they were. Data extracted from the systematic reviews also included information on the delivery of the intervention (eg, smartphone, web based, wearable activity tracker–only interventions, or mixed delivery methods), human and technological components included, and the duration of the interventions.

### Methodological Quality

The quality of the included systematic reviews was assessed using A Measurement Tool to Assess Systematic Reviews-2 (AMSTAR-2). This tool comprises 16 questions that are used to assess the confidence of the results of the review. The AMSTAR-2 rating of a review can range from high (when a review paper has <1 critical weakness) to moderate (with >1 noncritical weakness), low (with 1 critical weakness), and critically low (with >1 critical weakness) [28]. It is recommended that the ratings of the individual items should not be combined to create an overall rating, instead users should consider the impact of each individual item to determine an overall confidence rating.

All 16 questions within the AMSTAR-2 tool are of importance; however, there are 7 critical domains that can affect the validity of a review and the conclusions that can be drawn [28]. These critical domains relate to the review methodology, literature search strategy, justification for excluded studies, techniques for assessing the risk of bias, methodology for the statistical combination of results if meta-analysis was used, accounting for the risk of bias when discussing the results, and investigating publication bias and discussing the impact on results of the review, if quantitative synthesis was performed.

The methodological quality of each included systematic review was assessed by the main reviewer. The second reviewer assessed the methodological quality of 1 included review individually to cross-check the quality assessment process. Any disagreements or conflicts were resolved through discussion.

### Presentation of Findings

The results of this umbrella review provided a summary of systematic reviews assessing digital PA and SB interventions for community-living adults. These results were reported as a summary of the synthesized results. Findings were summarized to identify if interventions have been beneficial or effective, if no differences between interventions and control groups have been identified or mixed results, or to show if interventions were less effective than the comparator or not effective. These summaries can be used to easily understand if a statistically significant change in PA or SB was achieved.

A quantitative summary of the included systematic reviews in relation to their delivery methods and their content (inclusion of theoretical frameworks and BCTs) was also created. The technological delivery methods were split into 6 columns consisting of web-only delivery, smartphone- or tablet-only delivery, video game–only delivery, virtual reality–only delivery, monitor- or sensor-only delivery (including wearable activity monitors, pedometers, accelerometers, and heart rate sensors), and a mixed technological delivery column. The tally of these technological delivery components matched the number in the included studies column of the table. If a systematic review mentioned the use of theoretical frameworks and BCTs, they were marked “yes” with the number of mentioned frameworks and BCTs included in brackets.

## Results

### Study Inclusion

Academic database and gray literature searches identified 361 and 73 articles for screening, respectively (refer to Multimedia Appendix 1 for full search strategy and outputs). These 434 articles were exported into Endnote Online for initial screening, where 104 (24%) duplicates were removed. After this process, the remaining articles (n=330, 76%) were exported to the screening website Rayyan for title and abstract screening.

**Figure 1:**
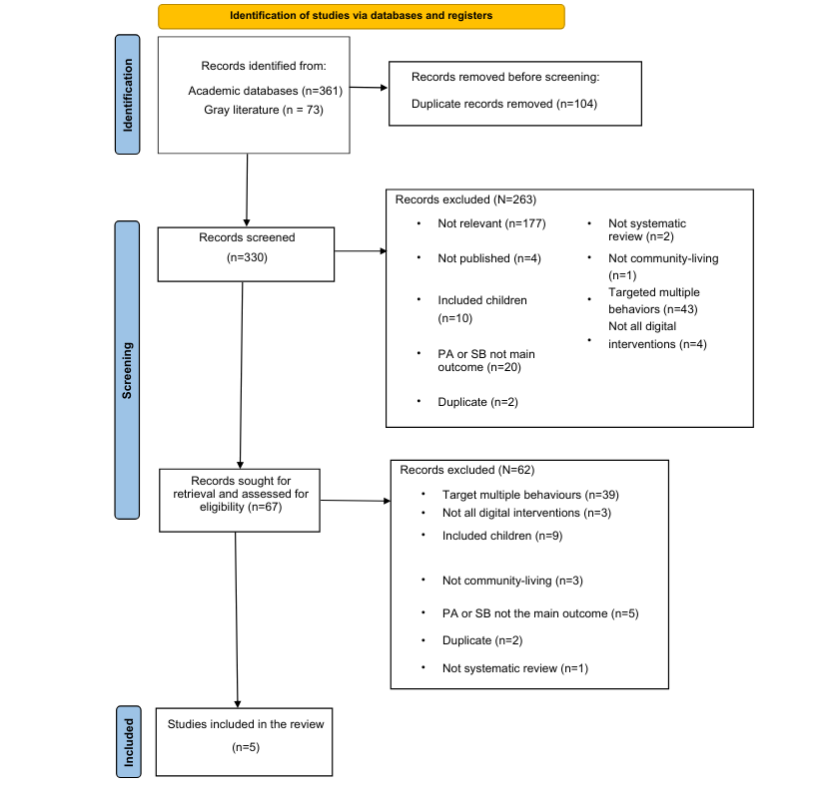
PRISMA (Preferred Reporting Items for Systematic Reviews and Meta-Analyses) diagram of the selection process. PA: physical activity; SB: sedentary behavior.

Title and abstract screening removed 263 (79.7%) of the 330 articles. The reasons for removal are displayed in Figure 1. With an agreement of 84%, the interrater reliability of title and abstract screening indicated substantial agreement between the main reviewer and second reviewer (WH; Cohen κ=0.69; *P*<.001). Conflicts (41/330, 12.4%) were resolved through discussions and clarification of the protocol.

After title and abstract screening, 67 articles were eligible for full-text screening. Screening of these articles was conducted in Rayyan with an additional Microsoft Excel spreadsheet used to track decisions. A total of 62 (93%) articles were removed during the screening process (Figure 1). There was 69% agreement between the main reviewer and additional reviewers for full-text screening, corresponding to a fair agreement (Cohen κ=0.25; *P*=.02). All conflicts (21/67, 31%) were resolved through discussions. The final full-text screening resulted in 5 systematic reviews to be included within this umbrella review [29-33].

### CCA Scores

The measured overlap of primary studies included in the eligible systematic reviews was measured to be 0% (Multimedia Appendix 2 [29-33]). This indicates that all included systematic reviews consisted of different primary studies.

### Characteristics of the Included Studies

A summary of the characteristics of the included systematic reviews is displayed in Table 1. The total number of primary studies included in the 5 systematic reviews represented 96 original research papers conducted across 24 different countries. The included systematic reviews’ publication dates ranged from 2018 [30] to 2023 [31,33]. The research designs varied within each systematic review and included randomized controlled trials, developmental studies, pre- and postintervention studies, and implementation studies. In total, 2 (40%) of the 5 systematic reviews included meta-analysis [31,33].

**Table 1:** Characteristics of the included systematic reviews.

Study	Countries of the studies	Papers reviewed and publication date range	Population	Sample size	The type of study included
Lee et al [29], 2023	Australia, Iran, Ireland, Japan, Spain, and the United States	18 (2010-2020)	Adults with obesity or adults who were overweight	Range 26-1755	RCT^a^ and pre-post study without a control group
Berry et al [30], 2018	Australia, Canada, the Netherlands, the United Kingdom, and the United States	9 (2006-2017)	Individuals with “arthritis” and those with chronic conditions	Range 20-968	RCT, pre-post test, 2-group pre-post test, and implementation study
Wang and Kassavou [31], 2023	Australia, Canada, China, Germany, Israel, Japan, Korea, the Republic of Slovenia, Spain, Taiwan, Thailand, Turkey, the United Kingdom, and the United States	16 (2008-2021)	Adults with a history or explicit diagnosis of stroke	799	RCT
Daryabeygi-Khotbehsara et al [32], 2021	Italy, Netherlands, New Zealand, and the United States	8 (2015-2020)	Insufficiently active and sedentary healthy adults, healthy and highly educated young adults, overweight and sedentary adults, adults with chronic low back pain, and students and staff from a university setting	Range 10-104	Pre-post intervention; RCT; 3-arm quasi-experimental, single-group micro randomized trial; and developmental study
Wu et al [33], 2023	Australia, Belgium, Canada, Chile, China, Denmark, Finland, Germany, Japan, Netherlands, Portugal, Spain, the United Kingdom, and the United States	45 (2003-2022)	Individuals living with chronic diseases and other diseases	Range 15-1023	RCT

^a^RCT: randomized controlled trial.

### Participant Characteristics

The sample sizes within the systematic reviews ranged from a single-group microintervention study consisting of 10 participants to a randomized controlled trial with 7144 participants. A total of 4 (80%) of the 5 systematic reviews described participants’ ages, which ranged from 19 to 89 years. Most systematic reviews (4/5, 80%) reported mixed sexes of participants, but there was a greater proportion of female participants in 2 (40%) of the systematic reviews.

### Intervention Characteristics

#### Overview

Of the 5 included systematic reviews, 2 (40%) reviews included interventions that only focused on improving PA [29,30], while the remaining systematic reviews included PA and SB interventions. The duration of interventions ranged from 2 weeks to 12 months.

#### Research Question 1: Effectiveness of Interventions

All (5/5, 100%) systematic reviews reported on changes in PA where there was a mixture of approaches, and 2 (40%) systematic reviews reported statistical significance of results. Both systematic reviews that included meta-analysis reported statistically significant changes in PA. Wu et al [33] reported significant increases in daily steps from 38 analyzed studies (standardized mean difference [SMD]=0.59; 95% CI 0.44-0.75; *P*<.001), total daily PA from 4 studies analyzed (SMD=0.21; 95% CI 0.01-0.40; *P*=.04), and time spent in MVPA from 18 analyzed studies (SMD=0.54; 95% CI 0.36-0.72; *P*<.001). Wang and Kassavou [31] found significant improvements from 10 studies analyzed, which included 326 participants, to support the use of digital interventions for increasing PA (SMD=0.39; 95% CI 0.17-0.61; *P*<.001; Table 2).

**Table 2:** Effectiveness of interventions.

Study	Meta-analysis	PA^a^ or SB^b^ only	PA or SB outcome assessment	Common PA outcomes					Common SB outcomes
				Steps^c^	MVPA^d^	Total PA^e^	PA change^f^	SB minutes or change	
Lee et al [29], 2023	No	PA only	Self-report and device based	—^g^	—	—	Mixed results	N/A^h^	
Berry et al [30], 2018	No	PA only	Self-report and device based	Mixed results	Mixed results	Mixed results	—	N/A	
Wang and Kassavou [31], 2023	Yes	PA and SB	Self-report and objective measurement	—	—	—	Statistically significant improvement	Statistically nonsignificant change	
Daryabeygi-Khotbehsara et al [32], 2021	No	PA and SB	Unclear	Mixed results	Statistically nonsignificant change	Statistically nonsignificant change	—	Statistically nonsignificant change	
Wu et al [33], 2023	Yes	PA and SB	Objective	Statistically significant improvement	Statistically significant improvement	Statistically significant improvement	—	Statistically significant improvement	

^a^PA: physical activity.

^b^SB: sedentary behavior.

^c^Steps included accelerometer, mean number of steps per day, step counts, average daily steps, daily walking time, daily step count, or daily steps.

^d^Moderate-to-vigorous PA (MVPA) outcomes included moderate PA minutes per week, MVPA time, average daily minutes MVPA, or MVPA minutes.

^e^Total PA outcomes included total PA minutes per week, total PA time, or total daily PA.

^f^PA change outcomes included PA or PA behavior.

^g^Not available.

^h^N/A: not applicable.

For 3 (60%) of the 5 systematic reviews that did not include meta-analysis, the effectiveness of digital interventions on PA was mixed (Table 2). Lee et al [29] reported 14 of the 18 interventions had a positive effect on PA; however, 4 interventions were reported to have “no effect” on PA. Similarly, Berry et al [30] found 6 studies reported significant increases in PA, but 3 studies reported nonsignificant improvements. Finally, Daryabeygi-Khotbehsar et al [32] reported significant changes in light-intensity PA, average daily steps, mean daily step time, and daily walking time. However, this systematic review also reported nonsignificant changes in daily minutes of MVPA and daily step counts.

Of the 5 included reviews, findings for the effectiveness of digital interventions to improve SB were also mixed across the 3 (60%) reviews reporting this behavior. The meta-analysis conducted by Wu et al [33] included 11 studies analyzing the effect of digital interventions on improving SB and found a small but favorable effect size for improving sedentary time by reducing time spent sedentary using digital interventions (SMD=–0.10; 95% CI –0.19 to –0.01; *P*=.03). However, 1 (20%) systematic review with meta-analysis, analyzing 11 studies, and 1 (20%) systematic review without meta-analysis, reported Statistically nonsignificant changes in SB (SMD=–0.13, 95% CI –0.31 to 0.05, N=473, *P*=.53 [31] and adjusted mean difference –9.5 min, SE 7.5; 95% CI 19.98-1.05; *P*=.08 [32]).

In answer to the first research question, digital PA interventions have the potential to improve PA outcomes. Of the 5 included systematic reviews used in this umbrella review, 2 (40%) undertook meta-analysis, which found statistically significant improvements in daily steps, minutes spent in MVPA, total PA time, and PA behavior. However, these findings were not universally true across the included systematic reviews of this umbrella review as the remaining included reviews, which did not include meta-analysis, reported mixed or statistically nonsignificant results across these outcomes. Findings from this umbrella review highlighted that the effectiveness of digital SB interventions was also contrasting. In addition, 1 (20%) systematic review with meta-analysis reported statistically significant improvements in SB through reduced sedentary time (min), but the other systematic review with meta-analysis and one other systematic review found statistically nonsignificant improvements in SB.

#### Research Question 2: Intervention Components and Content

##### Intervention Delivery

Both systematic reviews that included PA-only interventions used a range of technological delivery methods, including web-only methods (such as virtual avatars), smartphone- or tablet-only methods (including smartphone apps), monitor- or sensor-based methods (such as commercial grade accelerometers), and a mixed technological delivery method of a smartphone app linked to a wearable activity tracker (Table 3). The human components used in these interventions included feedback provided by trained moderators, private messaging, and video and audio communication between participants and moderators.

**Table 3:** Quantitative summary of components and content of included reviews.

Study	Included studies, (n=96), n (100%)	Web only, (n=15), n (16%)	Smartphone or tablet only, (n=10), n (11%)	Video game only, (n=5), n (5%)	Virtual reality only, (n=4), n (4%)	Monitor or sensor only, (n=36), n (39%)	Mixed technological intervention, (n=22), n (24%)	Theoretical framework (number identified)	Behavior change technique (number identified)
Lee et al [29], 2023	18 (19)	11 (61)	3 (17)	0 (0)	0 (0)	4 (22)	0 (0)	Yes (5)	Yes (11)
Berry et al [30], 2018^a^	9 (9)	4 (80)	—^b^	0 (0)	0 (0)	—	1 (20)	Yes (1)	Yes (15)
Wang and Kassavou [31], 2023	16 (17)	0 (0)	3 (19)	5 (31)	4 (25)	3 (19)	1 (6)	No (N/A^c^)	No (N/A)
Daryabeygi-Khotbehsara et al [32], 2021	8 (8)	—	4 (50)	0 (0)	0 (0)	—	4 (50)	Yes (8)	Yes (7)
Wu et al [33], 2023	45 (47)	—	—	0 (0)	0 (0)	29 (64)	16 (36)	No (N/A)	Yes (5)

^a^Berry et al [30] included 9 studies evaluating 5 different interventions; therefore, the tally of intervention delivery components will equal 5.

^b^Not available.

^c^N/A: not applicable.

For the 3 (60%) of the 5 systematic reviews that targeted both PA and SB, the technological components consisted of a variety of smartphone- and tablet-only interventions, video game–based interventions, virtual reality interventions, monitor- and sensor-based interventions, and a range of mixed technological delivery methods. Wang and Kassavou [31] reported 1 mixed intervention of a mobile phone and tablet intervention with video game components. Daryabeygi-Khotbehsara et al [32] reported 4 mixed interventions, 2 of which were smartphone apps with PA monitors or sensors (heart rate wristbands and Fitbit One [Google LLC]), 1 was a smartphone app with Fitbit and web-based mobile questionnaire, and 1 was a mixed intervention that did not mention the operating system but consisted of text messages and activPAL 3 (PAL Technologies Ltd) activity tracker. Finally, Wu et al [33] reported 16 mixed interventions, 4 of which were web based and monitor or sensor interventions and 12 which were smartphone and tablet and monitor or sensor mixed interventions (Table 3). The human components used in these interventions included manual logging of activity, PA consultations, nurse consultations, group education, training courses, and activity books. Only 1 (20%) systematic review [31] did not report on the human components used within the interventions.

##### Theoretical Frameworks

Of the 5 included systematic reviews, 2 (40%) reviews that included only PA interventions [29,30] and 1 (20%) systematic review that included PA and SB interventions [32] reported the use of theoretical frameworks to guide the interventions (Table 3). However, 1 (20%) of these systematic reviews [30] only reported on 1 framework for guiding the interventions and did not explicitly report on the use of multiple theoretical frameworks or gave details on other frameworks used.

Of the 5 systematic reviews, 3 (60%) that reported the use of theoretical models within interventions reported that social cognitive theory was the most prominent theory and guided most interventions. The frameworks reported by Lee et al [29] and Daryabeygi-Khotbehsara et al [32], included self-regulation theory (n=2), computational agent model (n=1), control theory (n=1), exploit-explore strategy (n=1), Fogg behavior model (n=1), health action process approach (n=1), health belief model (n=1), learning theory (n=1), self-efficacy theory (n=1), and transtheoretical model (n=1).

##### Behavior Change Techniques

A total of 4 (80%) of the 5 included systematic reviews [29,30,32,33] reported on the use of BCTs to support interventions (Table 3), and 3 (75%) of the 4 systematic reviews [29,30,32] reported the most frequently used BCTs to be goal setting, self-monitoring, feedback, and social support. Aside from the most frequently used BCTs, the additional BCTs identified from the 4 systematic reviews include information on health consequences (2/28, 7%), action planning, body changes (1/28, 4%), the Coventry, Aberdeen, and London–Refined taxonomy of BCTs (1/28, 4%), culturally relevant information (1/28, 4%), discussion about incompatible beliefs (1/28, 4%), distraction (1/28, 4%), emotional support (1/28, 4%), framing and reframing (1/28, 4%), habit formation (1/28, 4%), information on how to perform PA (1/28, 4%), past activity performances (1/28, 4%), PA advice (1/28, 4%), problem-solving (1/28, 4%), prompts and cues (1/28, 4%), review of goals (1/28, 4%), reviewing behavior (1/28, 4%), reward (1/28, 4%), self-efficacy (1/28, 4%), self-identity (1/28, 4%), self-talk (1/28, 4%), social modeling (1/28, 4%), theory-based strategies (1/28, 4%), and watching videos (1/28, 4%).

Only 1 (20%) of the 5 systematic reviews did not report on the inclusion of BCTs used within PA and SB interventions [31]. However, it did report on the effectiveness of self-monitoring and behavior change. For changes in both PA and SB, interventions underpinned by self-monitoring elements were more likely to explain effectiveness. In relation to PA, self-monitoring interventions had a moderate to large impact on improving behavior (SMD=0.56; 95% CI 0.21-0.91; *P*=.002). Similarly, SB self-monitoring interventions significantly explained the overall effectiveness of reducing sedentary time compared to those not including such components (SMD=–0.34; 95% CI –0.65 to –0.03; *P*=.03).

In answer to the second research question, of the 5 included systematic reviews used in this umbrella review, 4 (80%) reported the use of human elements within interventions [29,30,32,33], all 5 (100%) reported the digital elements used to deliver the interventions, 3 (60%) reported the use of theoretical frameworks to guide interventions, and 4 (80%) reported the use of BCTs to support the interventions. Monitor- and sensor-only intervention delivery methods were used most frequently followed by mixed technological delivery methods. In total, 11 different theoretical frameworks were identified from the systematic reviews, with social cognitive theory reportedly being the most prominent theory used to guide interventions. In addition, 28 different BCTs were reported in the systematic reviews, with goal setting, self-monitoring, feedback, and social support being reported as the most frequently used BCTs. Moreover, 1 (20%) systematic review also reported that self-monitoring was more likely to explain improvements in both PA and SB compared to interventions that did not include this BCT.

#### Methodological Quality

The methodological quality of all included systematic reviews was assessed by the main reviewer, and the quality assessment process of the main reviewer was checked by the second reviewer. This quality assessment of the process resulted in moderate agreement (69%; Cohen κ=0.43; *P=*.04).

The use of the AMSTAR-2 tool resulted in 20% (1/5) low-quality systematic reviews [32] and 80% (4/5) critically low–quality systematic reviews [29-31,33] (Table 4). The common methodological strengths of the included reviews (common in ≥3 systematic reviews) were the inclusion of Population, Intervention, Comparison, and Outcome components, reporting that the review methods were established before the conduct of the review, the explanation of study design, descriptions of the included studies, using a satisfactory technique for assessing the risk of bias in individual studies, accounting for the risk of bias when interpreting results, and reporting any potential conflicts of interest. The common areas of weakness (present in ≥3 systematic reviews) in noncritical domains were not performing study selection in duplicate, not performing data extraction in duplicate, not reporting the sources of funding for the primary studies included in the review, and not providing a satisfactory explanation and discussion of any heterogeneity observed in the results of the review. The common areas of weakness in critical domains were not using a comprehensive literature search strategy and not providing a list of excluded studies and the justification for the exclusions.

**Table 4:** Methodological quality of included systematic reviews.

Study	Noncritical domain strengths, n	Critical domain strengths, n	Noncritical domain weaknesses, n	Critical domain weaknesses, n	AMSTAR-2^a^ rating
Lee et al [29], 2022	6	3	2	2	Critically low
Berry et al [30], 2018	4	1	4	4	Critically low
Wang and Kassavou [31], 2023	5	3	4	4	Critically low
Daryabeygi-Khotbehsara et al [32], 2021	3	4	5	1	Low
Wu et al [33], 2023	4	5	5	2	Critically low

^a^AMSTAR: A Measurement Tool to Assess Systematic Reviews-2.

## Discussion

### Principal Findings

This umbrella review synthesized evidence from 5 systematic reviews exploring digital PA and SB interventions for community-living adults. Social cognitive theory, goal setting, self-monitoring, feedback, and social support were found to be the most prominent theoretical frameworks and BCTs used in digital interventions. Overall, the systematic reviews reported contrasting findings for digital interventions, with some reviews reporting statistically significant improvements in PA and others reporting mixed effects for improvements in PA. Similarly, evidence regarding the effectiveness of digital interventions for SB was mixed, as only 1 (20%) systematic review reported statistically significant reductions in sedentary time, while 2 (40%) systematic reviews reported no significant changes in SB. Finally, the included systematic reviews had 0% CCA overlap, but they were of low and critically low quality.

### Effectiveness to Improve PA and SB

Digital interventions had the potential to significantly improve PA outcomes; however, findings from this umbrella review were mixed. A total of 3 (60%) of the 5 systematic reviews comprised mixed results to support the effectiveness of interventions to significantly improve PA. The 2 (40%) systematic reviews that reported statistically significant results for changes in PA consisted of smartphone or tablet, video game, virtual reality, monitor- or sensor-based interventions, and mixed technological delivery methods. Surprisingly, these systematic reviews did not report on the theoretical basis of the interventions, and only 1 (20%) systematic review discussed the use of BCTs within the interventions.

The effectiveness of interventions targeting SB varied across the included systematic reviews. While one systematic review suggested the use of digital interventions and had small but significant reductions in sedentary time, other systematic reviews found nonsignificant effects on SB. There were some differences between the intervention delivery methods of the significant and nonsignificant systematic reviews, which could explain the differences in success for changing SB, for example, of the 5 included reviews, 2 (40%) nonsignificant systematic reviews had a lower proportion of monitor- or sensor-based and mixed technological delivery methods. However, these are observations. It could be argued that the challenge in measuring PA and SB outcomes is heavily dependent on the metrics used. Previous work aiming to compare movement behavior features processed by commonly used accelerometer metrics among adults found statistically significant differences in 24-hour movement behavior across 4 different metrics used to measure these behaviors [34]. Therefore, suggesting the success of intervention may be overestimated or underestimated depending on the metrics used to measure these PA and SB outcomes, thus resulting in different results and conclusions.

In addition, although most changes to SB were nonsignificant, reductions in SB were reported. Research has found that breaking up SB with short bursts of light-intensity PA can improve metabolic health and self-reported fatigue and reduce all-cause mortality [35]. Therefore, these interventions can still be beneficial for health, but the mixed evidence suggests that further research is needed to determine the most effective strategies to include within digital interventions to reduce SB within community-living adults.

Traditionally, public health research has focused on the importance of increasing PA to improve health and, as a result, there are more interventions to improve PA. However, as worldwide levels of SBs have risen in recent years [36] and more information about the health consequences of this has been recognized, the interest in SB has grown, but the development of interventions and subsequent research exploring effectiveness is still emerging. The substantial variation in how SB is defined across accelerometer brands and studies underscores the evolving nature of SB research, interventions, and commercial products [37]. The differences in the effectiveness of SB interventions within this umbrella review may be due to the systematic reviews and primary studies within them using different technologies and terminologies to understand SB. Once again, this highlights the difficulty in interpreting whether the interventions were effective or not.

Although findings are inconclusive regarding the effect of digital interventions on SB, this research has revealed that the inclusion of self-monitoring is more likely to explain changes in behavior in favor of the intervention. Of the 5 included systematic reviews, 1 (20%) reported that interventions consisting of self-monitoring components had a moderate-to-large impact on positively changing PA behavior, and although the change in SB was reported as not statistically significant, self-monitoring interventions were more effective in changing SB than those without this component.

Previously published systematic reviews have described self-monitoring to be a valuable and effective instrument for encouraging positive changes in PA behaviors [38,39]. Accurate self-monitoring of PA and SB has the potential to increase individuals’ awareness of their behavior, which can encourage them to change their habits toward PA and SB. Self-monitoring through digital devices can also provide detailed data on individuals’ movement behaviors and help detect patterns and triggers of these behaviors. Allowing tailored feedback based on these data can also enhance the effectiveness of this type of intervention [40].

Although research suggests the use of self-monitoring for effective behavior change, evidence of effectiveness for this BCT has been found to vary significantly across different populations and health behaviors [41]. To achieve more effective changes in PA and SB outcomes, self-monitoring needs to be used consistently [42]. Therefore, this highlights the need for research into the use of this BCT element in different populations, including informal carers, and for different health behaviors for it to be implemented effectively to create successful interventions, including digital interventions, to improve PA and reduce SB.

### Theoretical Frameworks and BCTs

Findings from this umbrella review regarding the most prominent theoretical framework and implemented BCTs have been supported by previous literature. A systematic review and meta-analysis aimed to identify the most effective BCTs for increasing PA in digital and face-to-face interventions in adults with obesity or adults who are overweight reported that 45 included intervention studies were grounded in one or multiple theoretical frameworks [43]. Social cognitive theory and self-regulation theory, alone or in combination, were used most often in digital interventions aimed at increasing PA [43]. A scoping review of user models for personalized PA interventions also revealed prominent theoretical frameworks used in interventions, including Fogg behavior model, social cognitive theory, transtheoretical model, and health action process approach [44], all of which were identified within this umbrella review. Finally, scoping reviews have also reported that the most frequently implemented BCTs in digital PA interventions for adults include goal setting, self-monitoring, and feedback on behaviors [45,46].

Although there are similarities between the findings of this umbrella review and previous work, other theoretical frameworks and BCTs have been reported in these studies that were not reported in this umbrella review. Ghanvatkar et al [44] mentioned that the theory of planned behavior, the I-change model, and the behavior change wheel were also found to frequently guide digital PA interventions [44]. Similarly, De Santis et al [45] and Meinhart et al [46] found that providing educational and motivational content, feedback on exercise, and integrated tailored responses were all present in digital interventions. In addition, a systematic review exploring the effectiveness of BCTs at increasing older adults’ self-efficacy and PA reported that using barrier identification and problem-solving, providing rewards contingent on successful behavior, and demonstrating the behavior were significantly associated with higher levels of PA [47]. It also reported that the use of providing normative information about others’ behavior, providing information on where and when to perform the behavior, and planning social support or social change resulted in significantly lower PA behavior effect sizes for older adults [47].

While there is overlap between the findings of this umbrella review and previous research regarding the theoretical frameworks and BCTs used in digital PA interventions, there are additional frameworks and BCTs not reported in this umbrella review, highlighting the diversity of these interventions for promoting and improving PA and SB levels. The reason for these differences may be due to the different populations targeted and the different interventions used. However, it is also important to consider that not all interventions will use the same frameworks or BCTs nor will they recognize and report on all frameworks or BCTs used. Intervention descriptions may not have been sufficiently detailed and precise enough to identify the theoretical frameworks and BCTs present within primary studies or systematic reviews. With regard to this umbrella review, the identification of the implemented theoretical frameworks and BCTs was limited to their presentation within the systematic reviews used for this research. Therefore, our findings are subject to the parameters of the systematic reviews and not the primary studies reported within them.

It is also important to note that just because certain frameworks and BCTs were not reported, it does not mean they are not used or worthwhile being used; it is simply that they were not reported to be present in the systematic reviews included in this umbrella review. There are many other theoretical frameworks and BCTs that could potentially be of use, specifically for different populations, as demonstrated by the other reviews discussed. However, more research is needed to understand the effectiveness of these theories and BCTs, especially within the informal caring population.

### Quality of Included Reviews

The quality assessment process, which showed moderate agreement between reviewers, highlights the credibility of the quality assessment process. However, the methodological quality of the included systematic reviews was low (20%) and critically low (80%).

While there were some common strengths of the systematic reviews regarding methodological quality, for example, the inclusion of Population, Intervention, Comparison, and Outcome components, reporting that the review methods were established before the conduct of the review and the explanation of study design can strengthen the methodological rigor of these systematic reviews. The methodological weaknesses, such as a lack of a comprehensive literature search strategy and a lack of justification for excluded studies, could reduce the confidence in the validity of the included systematic reviews.

The low confidence ratings of these systematic reviews could be because of the high standards set by the AMSTAR-2 tool. Although AMSTAR-2 has been recognized as a useful appraisal tool to understand the quality of published systematic reviews [48], there are some challenges in using this method for quality assessment. It has been discussed in previous work and reported in other umbrella reviews that AMSTAR-2 produces a generally high proportion of critically low confidence ratings among systematic reviews of health interventions, and there is ongoing debate whether this is due to poor discriminatory powers of the tool or the low quality of systematic reviews of health care interventions [49]. Individual items of AMSTAR-2 have been discussed in work by De Santis et al [49] to highlight difficulties of the tool and potential reasons for lower quality ratings of systematic reviews, for example, the “all-or-none” ratings of items that do not allow to differentiate between missing information and incomplete reporting of information for some items. The agreement of 69% between reviewers for approaching AMSTAR-2 in this umbrella review highlights the subjectiveness of this methodological tool, which has also been criticized by De Santis et al [49]. Due to the rapid increase in systematic reviews for health care interventions, it has been recommended that the items of AMSTAR-2 be empirically tested to improve the appraisal process and increase user consensus.

As this umbrella review explores digital interventions for improving PA and SB for community-living adults, its findings may have practical implications for public health policies and future digital interventions. However, as findings are from low and critically low–quality systematic reviews, there is a need for cautious interpretation. Stakeholders and policy makers must be aware of the methodological limitations of these systematic reviews when considering the findings presented within this umbrella review. Furthermore, these findings suggest the importance and need for more high-quality research within this field to provide methodologically sound evidence for future policy makers and decision makers.

### Strengths

There are multiple strengths of this umbrella review. First, this umbrella review followed the Joanna Briggs Institute framework, ensuring a rigorous and systematic approach. Adherence to this framework also promotes transparency and replicability, making the results more robust and credible. The incorporation of a large number of primary publications from the included systematic reviews allowed a broad and inclusive scope of this review to be conducted. All primary publications were unique with a CCA score of 0%, which indicates no overlap and strengthens the robustness of the review’s conclusions. Furthermore, the geographic reach of the reviews included and the primary studies within them were extensive, with studies conducted in 24 different countries. This wide geographic representation adds to the generalizability of the findings across diverse populations and different groups of community-living adults. Another strength of this review was the identification of a vast range of theoretical frameworks and BCTs, which provides a comprehensive understanding of the strategies used in digital PA and SB interventions for community-living adults. Finally, the search strategy of this review highlights a critical gap in the existing literature as very few systematic reviews focused purely on PA and SB and theoretical frameworks and BCTs, as numerous systematic reviews had to be excluded due to their inclusion of multiple behaviors, such as diet and sleep.

### Limitations

The lack of high-quality systematic reviews within this umbrella review restricts the potential to use these findings to aid future decision-making. Therefore, there is a need for more high-quality systematic reviews in the digital PA and SB intervention research field that include justifications for the search strategy, provide lists of excluded studies, report the sources of funding for the included studies, and provide an explanation for heterogeneity. As most (3/5, 60%) included systematic reviews lacked meta-analysis and reported multiple different PA outcomes, it is difficult to synthesize quantitative data and nonquantitative data to provide a clear answer to understand the effectiveness of these interventions in improving PA and SB. Finally, it is possible that some relevant studies were missed due to differences in the search strategies, inclusion criteria, and the databases used. Furthermore, restricting the review to English-language publications may have excluded important research published in other languages.

### Future Work

Future work should include more primary studies and systematic reviews to be published specifically focusing on PA- and SB-only interventions instead of interventions that combine these behaviors with other health behaviors, such as diet and sleep. There is also a need for more primary studies to use consistent and accurate measures of PA and SB, and standardized outcomes for measuring these behaviors should be implemented.

In addition, further investigation is also warranted for the development and refinement of intervention strategies specifically for reducing SB among community-living adults to achieve more robust outcomes of the effectiveness of digital interventions to reduce this behavior. Recommendations for future work also include better reporting of theoretical frameworks and BCTs used in digital interventions for PA and SB and meta-analysis of currently published systematic reviews to allow for further evaluation to be conducted into the effectiveness of these components to change behaviors. Finally, more meta-analyses of systematic reviews assessing the effectiveness of digital interventions on PA and SB are needed to provide a more comprehensive and accurate picture of the current evidence.

### Conclusions

This umbrella review aimed to evaluate the effectiveness of digital interventions for changing PA and SBs among community-living adults, identify the components and content of these interventions, and assess the methodological quality of the included systematic reviews.

Overall, this umbrella review indicates that digital interventions have the potential to improve PA and reduce SB. Meta-analyses suggest these interventions can be effective, particularly those incorporating self-monitoring and BCTs. However, more work is needed to understand the effectiveness and impact of specific theoretical frameworks and BCTs on changing PA and SBs. The mixed findings and methodological weaknesses highlight the need for more comprehensive reporting and analyses to be conducted to understand the effectiveness of digital interventions for community-living adults.

In relation to informal carers, this umbrella review highlights that digital PA and SB interventions offer a valuable opportunity to support behavior change in this population due to their scalability and numerous delivery methods. These digital interventions have been found to consist of a theoretical grounding and can incorporate numerous BCTs, all of which could improve the effectiveness of these interventions to support and encourage PA and reduce SB in informal carers. Despite the potential benefits of these interventions for informal carers, more coordinated efforts and research are needed to understand the potential and effectiveness of digital interventions for improving PA and reducing SB in this group.

## Appendix

Multimedia Appendix 1 Search strategy, keywords, and outputs.

Multimedia Appendix 2 Overlap of reviews.

## Abbreviations

AMSTAR-2: A Measurement Tool to Assess Systematic Reviews-2
BCT: behavior change technique
CCA: corrected covered area
DHI: digital health intervention
MVPA: moderate-to-vigorous physical activity
PA: physical activity
PICOS: population, intervention, context, outcomes, and study
SB: sedentary behavior
SMD: standardized mean difference
