# Supplementary material for: Digital Physical Activity and Sedentary Behavior Interventions for Community-Living Adults: Umbrella Review
Source: J Med Internet Res. 2025 Mar 18;27:e66294. doi: 10.2196/66294 (PMC11962315; doi:10.2196/66294)
Supplement: Multimedia Appendix 1 [file jmir_v27i1e66294_app1.doc]

*Appendix A: Keywords & Search strategy*

| Search No. | Topic | Keywords | Output |
| --- | --- | --- | --- |
| 1 | Adult | Adult* | Web of Science: 2,031,855  Cochrane: 3,605  PROSPERO: 76,350  MEDLINE (Ovid): 6,348,028  APA PsychINFO: 507,155  CINAHL: 460,702  INSPEC: 43,529  ARXIV: 1,572  CORE: 5,031,271  OPENGREY: 2,757  OPEN DISSERTATIONS: 51,449 |
| 2 | Digital | digital OR mobile OR smartphone OR mHealth OR “mobile health” OR eHealth OR “electronic health” OR “fitness track*” OR “physical activity monitor” OR “wearable activity track*” OR “wearable activity monitor” OR Fitbit OR accelerometer OR “physical activity technolog*” OR “digital interventions” OR “digital heath intervention” OR “activity monitor” OR “wearable technolog*” OR “step counter” | Web of Science: 1,442,897  Cochrane: 372  PROSPERO: 10,702  MEDLINE (Ovid): 3354,172  APA PsychINFO: 66,291  CINAHL: 123,578  INSPEC: 1,803,756  ARXIV: 62,123  CORE: 1,362  OPENGREY: 18,571  OPEN DISSERTATIONS: 38,345 |
| 3 | Physical activity & Sedentary behaviour | “physical activity” OR PA OR exercise OR “physical exercise” OR “step count” OR “daily steps” OR “movement behaviour” OR “leisure time physical activity” OR “physical fitness” OR “sedentary behavio?r” OR SB OR sedentary OR inactivity OR “sedentary time” OR “sitting time” OR sitting OR “physical inactivity” OR deskbound | Web of Science: 1,099,819  Cochrane: 1,387  PROSPERO: 27,465  MEDLINE (Ovid): 665,981  APA PsychINFO: 138,338  CINAHL: 257,269  INSPEC: 215,650  ARXIV: 46,472  CORE: 22,015  OPENGREY: 28,837  OPEN DISSERTATIONS: 29,141 |
| 4 | Theoretical Frameworks & Behaviour Change Techniques | theoretical model” OR “behavio?r change” OR “behavio?r change technique” OR BCT OR “theoretical framework” OR “behavio?r change strateg*” OR “transtheoretical model” OR theory OR model | Web of Science: 13,600,908  Cochrane: 1,986  PROSPERO: 93,784  MEDLINE (Ovid): 3,179,262  APA PsychINFO: 1,226,679  CINAHL: 742,252  INSPEC: 8,197,864  ARXIV: 9,279  CORE: 7,179  OPENGREY: 23,916  OPEN DISSERTATIONS: 385,335 |
| 5 | Systematic Review | “systematic review” | Web of Science: 310,898  Cochrane: 1,933  PROSPERO: 206,387  MEDLINE (Ovid): 302,740  APA PsychINFO: 42,874  CINAHL: 280,798  INSPEC: 10,940  ARXIV: 3,360  CORE: 2,634,442  OPENGREY: 1,130  OPEN DISSERTATIONS: 5,730 |
| 6 | Adult AND Digital | ((adult*) AND (digital OR mobile OR smartphone OR mHealth OR “mobile health” OR eHealth OR “electronic health” OR “fitness track*” OR “physical activity monitor” OR “wearable activity track*” OR “wearable activity monitor” OR Fitbit OR accelerometer OR “physical activity technolog*” OR “digital interventions” OR “digital heath intervention” OR “activity monitor” OR “wearable technolog*” OR “step counter”)) | Web of Science: 40,358  Cochrane: 185  PROSPERO: 4,271  MEDLINE (Ovid): 67,201  APA PsychINFO: 7,125  CINAHL: 11,333  INSPEC: 5,293  ARXIV: 169  CORE: 1,346  OPENGREY: 32  OPEN DISSERTATIONS: 1,217 |
| 7 | Physical activity AND Theoretical Frameworks and Behaviour change techniques | ((“physical activity” OR PA OR exercise OR “physical exercise” OR “step count” OR “daily steps” OR “movement behaviour” OR “leisure time physical activity” OR “physical fitness” OR “sedentary behavio?r” OR SB OR sedentary OR inactivity OR “sedentary time” OR “sitting time” OR sitting OR “physical inactivity” OR deskbound) AND (“theoretical model” OR “behavio?r change” OR “behavio?r change technique” OR BCT OR “theoretical framework” OR “behavio?r change strateg*” OR “transtheoretical model” OR theory OR model)) | Web of Science: 209,675  Cochrane: 0  PROSPERO: 12,253  MEDLINE (Ovid): 85,930  APA PsychINFO: 33,524  CINAHL: 34,948  INSPEC: 57,751  ARXIV: 353  CORE: 1,199  OPENGREY: 0  OPEN DISSERTATIONS: 8,421 |
| 8 | Systematic review AND Adult AND Digital | ((“systematic review”) AND (adult*) AND (digital OR mobile OR smartphone OR mHealth OR “mobile health” OR eHealth OR “electronic health” OR “fitness track*” OR “physical activity monitor” OR “wearable activity track*” OR “wearable activity monitor” OR Fitbit OR accelerometer OR “physical activity technolog*” OR “digital interventions” OR “digital heath intervention” OR “activity monitor” OR “wearable technolog*” OR “step counter”)) | Web of Science: 1,389  Cochrane: 41  PROSPERO: 4,245  MEDLINE (Ovid): 1,211  APA PsychINFO: 247  CINAHL: 645  INSPEC: 59  ARXIV: 0  CORE: 1,132  OPENGREY: 3  OPEN DISSERTATIONS: 45 |
| 9 | Adult AND Digital AND Physical activity & sedentary behaviour AND Theoretical Framework & Behaviour Change Techniques AND Systematic review | ((adult*) AND (digital OR mobile OR smartphone OR mHealth OR “mobile health” OR eHealth OR “electronic health” OR “fitness track*” OR “physical activity monitor” OR “wearable activity track*” OR “wearable activity monitor” OR Fitbit OR accelerometer OR “physical activity technolog*” OR “digital interventions” OR “digital heath intervention” OR “activity monitor” OR “wearable technolog*” OR “step counter”) AND (“physical activity” OR PA OR exercise OR “physical exercise” OR “step count” OR “daily steps” OR “movement behaviour” OR “leisure time physical activity” OR “physical fitness” OR “sedentary behavio?r” OR SB OR sedentary OR inactivity OR “sedentary time” OR “sitting time” OR sitting OR “physical inactivity” OR deskbound) AND (“theoretical model” OR “behavio?r change” OR “behavio?r change technique” OR BCT OR “theoretical framework” OR “behavio?r change strateg*” OR “transtheoretical model” OR theory OR model) AND (“systematic review”)) | Web of Science: 134  Cochrane: 0  PROSPERO: 71  MEDLINE (Ovid): 86  APA PsychINFO: 25  CINAHL: 43  INSPEC: 2  ARXIV: 0  CORE: 61  OPENGREY: 0  OPEN DISSERTATIONS: 12 |
